# Advancing clinical leadership to improve the implementation of evidence-based practice in surgery: a longitudinal mixed-method study protocol

**DOI:** 10.1186/s13012-020-01063-2

**Published:** 2020-12-01

**Authors:** Amy Grove, Aileen Clarke, Graeme Currie, Andy Metcalfe, Catherine Pope, Kate Seers

**Affiliations:** 1 Health Technology Assessment and Implementation Science, Warwick Medical School, University of Warwick, Coventry, CV4 7AL UK; 2 Public Health and Health Services Research, Warwick Medical School, University of Warwick, Room B-162, Coventry, CV4 7AL UK; 3 Public Management, Warwick Business School, University of Warwick, Coventry, CV4 7AL UK; 4 Trauma and Orthopaedic Surgery, Warwick Medical School, University of Warwick, Coventry, CV4 7AL UK; 5 Medical Sociology, Nuffield Department of Primary Care Health Sciences, University of Oxford, Oxford, OX2 6GG UK; 6 Health Services Research, Warwick Medical School, University of Warwick, Coventry, CV4 7AL UK

**Keywords:** Orthopaedics, Leadership, Evidence-based practice, Social Network Analysis-Longitudinal Interviews, Qualitative

## Abstract

**Background:**

Clinical leadership is fundamental in facilitating service improvements in healthcare. Few studies have attempted to understand or model the different approaches to leadership which are used when promoting the uptake and implementation of evidence-based interventions. This research aims to uncover and explain how distributed clinical leadership can be developed and improved to enhance the use of evidence in practice. In doing so, this study examines implementation leadership in orthopaedic surgery to explain leadership as a collective endeavour which cannot be separated from the organisational context.

**Methods:**

A mixed-method study consisting of longitudinal and cross-sectional interviews and an embedded social network analysis will be performed in six NHS hospitals. A social network analysis will be undertaken in each hospital to uncover the organisational networks, the focal leadership actors and information flows in each organisation. This will be followed by a series of repeated semi-structured interviews, conducted over 4 years, with orthopaedic surgeons and their professional networks. These longitudinal interviews will be supplemented by cross-sectional interviews with the national established surgical leaders. All qualitative data will be analysed using a constructivist grounded theory approach and integrated with the quantitative data. The participant narratives will enrich the social network to uncover the leadership configurations which exist, and how different configurations of leadership are functioning in practice to influence implementation processes and outcomes.

**Discussion:**

The study findings will facilitate understanding about how and why different configurations of leadership develop and under what organisational conditions and circumstances they are able to flourish. The study will guide the development of leadership interventions that are grounded in the data and aimed at advancing leadership for service improvement in orthopaedics. The strength of the study lies in the combination of multi-component, multi-site, multi-agent methods to examine leadership processes in surgery. The findings may be limited by the practical challenges of longitudinal qualitative data collection, such as ensuring participant retention, which need to be balanced against the theoretical and empirical insights generated through this comprehensive exploration of leadership across and within a range of healthcare organisations.

Contributions to the literature

- We will extend the literature by exploring distributed leadership in healthcare—and position leadership as a collective social process which cannot be studied in isolation from the organisation in which it functions.
- We will advance the literature by revealing the configurations of leadership which appear to be effective for implementation across a range of healthcare contexts and demonstrate their links to service improvement and healthcare innovation.
- We will elucidate how, and the reasons why, effective implementation leadership works and potential mechanisms that can be implemented and tested in future research.

## Background

### Evidence-based service improvement in surgery

Healthcare services across the globe call for evidence-based service improvement and innovation [1]. The implementation and uptake of evidence-based interventions is an important part of driving service improvements [2]. However, there are prevailing gaps between what research evidence recommends and what frontline clinical professionals deliver [3]. This is enduring issue in surgery, where we have unwarranted variation in practice and economic pressures which cannot meet the increasing demand for services [4, 5]. The study of implementation science in surgery is a relatively young field of investigation. Little is known about the instrumental actors or the behaviors and actions of groups who promote the uptake of evidence-based interventions in surgical practice [6].

Much of the existing literature concentrates on the strategies of a particular individual, such as an ‘implementation leader’ or ‘champion’, and the powerful role they play in the implementation of evidence-based interventions [7–10]. Researchers have demonstrated the importance of clinical leadership in establishing service improvements and innovations—such as gaining institutional support for new practices, obtaining resources and building organisational partnerships [11–16]. However, leadership in healthcare implementation studies is often operationalised as a dichotomous state, as either present or absent—positive or negative [9]. Few studies have attempted to understand and model the different approaches to leadership that are used and which are most successful. Identifying and understanding the different configurations of leadership is necessary to describe and explain the mechanisms by which leadership can influence implementation processes and outcomes [17, 18].

### Leadership in surgery

In surgery, there is support for leadership roles in smaller surgical operating teams and across clinical departments, where leadership tends to constitute the behaviour of an individual surgeon [19, 20]. Studies revealed that individual surgeon leaders can have a large influence on the decision-making of surgical colleagues [21–23]. These individuals are highly visible and can improve communication between professional groups to facilitate practice decisions [24, 25]. Bonawitz and colleagues (2020) acknowledged the importance of understanding ‘who’ is leading implementation work specifically and subsequently developed the six attributes which they consider promote implementation success [26].

However, adopting this individualistic, attribute-focused explanation of leadership limits understanding about how and why implementation leadership works and the mechanisms needed for making improvements [27]. Equally, focusing on the characteristics of individual leaders cannot account for the potential interactions between leadership processes and organisational contexts—i.e., the set of interacting influences in clinical practice. What is missing in the literature is a detailed explanation of how and why different patterns of leadership differentially affect service improvement, and the types of leadership implementation strategies and interventions which could help foster implementation success in surgery.

A better understanding of leadership implementation efforts, as well as how and why leadership is effective and in what contexts, will help to facilitate more successful efforts to identify and advance clinical leadership in surgery. Filling this knowledge gap will identify the contextual influences and the mechanisms of evidence-based implementation which will be crucial to the development of targeted implementation interventions in the surgical specialties [28]. To achieve this depth of understanding, our study will examine surgical leadership across a range of hospital contexts to answer the following research question: How can clinical leadership be improved to enhance the use of evidence in orthopaedic surgical practice?

### Theoretical and conceptual framework: ‘leadership for implementation’

Implementation research has traditionally focused on the links between leadership styles and organisational outcomes [29, 30]. Yet, the theoretical foundations which bridge the empirical observations and implementation outcomes to the explanatory mid-range theories are lacking [28]. Establishing this theoretical underpinning is important for progressing the study of leadership and implementation of evidence-based practice. In this paper, we adopt the theory of distributed leadership (DL) as the conceptual and theoretical framework guiding our study [31–33].

Distributed leadership draws on more than 20 years of literature which shifted the focus from the attributes, behaviours and actions of individual ‘heroic’ leaders [34, 35], to contextualise leadership as a more collective social process which emerges through the interactions of multiple actors [36]. Fitzgerald et al. (2013) provided an empirically based definition of distributed leadership in healthcare, as a multi-professional organisation consisting of three elements spread across senior, middle and lower organisational levels [37]. We recognise that both individualistic and collective approaches to leadership are required in healthcare service delivery. The necessity of each, not only depends on the power and disposition of those involved, but also the contextual environment of the hospital. For example, individual leadership in a time of organisational crisis may be essential [38].

Our focus on DL, and particularly the leadership within surgical groups, extends our previous research to examine in more detail the collective dynamic of surgeons [39]. In our empirical study of guideline implementation, we revealed the importance attached to clinical leadership as an organisational process, in the mobilisation of evidence-based recommendations. We found that individual healthcare leaders often lacked status and position to achieve change within their organisations [23]. Here, we seek to uncover the legitimacy and disposition towards change that can be achieved by groups of surgeons and their professional network, working and leading as a collective. Fitzgerald and colleagues (2013) previously explored patterns of leadership in multi-professional healthcare organisations to understand relationships between patterns of DL and their impact on mandated service change [37]. Important for our study, are their theoretical contributions which suggest that strong pre-existing social relationships underpin the capacity of DL to implement service improvements [37]. This suggests conversely that poor relationships and conflict may potentially lead to poor implementation practices.

In this paper, we describe the construct of leadership for implementation, as a collective evidence-based implementation-related process undertaken through the interactions of multiple actors. We position leadership as the contextually situated process of a collective group, not the actions of an individual. From a distributed perspective, leadership practice forms through the interactions of groups of ‘leaders and followers’ and their contextual situation. Understanding context is important in the definition of DL, particularly in the field of implementation research where context plays a vital role in implementation success [40]. There is strong empirical evidence for the importance of leadership in predicting successful implementation interventions, but the majority focuses on transformational [41, 42] or transactional leadership [27, 43]. Few studies focused on how we can change leadership configurations to support implementation and the impact that developing and moving towards DL can have in surgical practice. The aim of our study is to understand and explain how distributed clinical leadership can be developed and improved to enhance the use of evidence in practice. In doing so, we focus specifically on the orthopaedic surgery to extend our previous exploratory research in this field [23, 39, 44].

## Methods

### Design

This mixed methods study seeks to understand and explain clinical leadership through an in-depth longitudinal qualitative examination and embedded social network analysis of leadership in the implementation of evidence-based service improvement and innovation. The study design contains two components which will be performed concurrently across six NHS hospitals. Initially, an embedded social network analysis will be performed at each hospital to reveal the organisational networks in the hospitals and how they are associated to leadership configurations and approaches to implementation. Second, a series of longitudinal interviews (over 4 years) with surgeon participants and their professional networks, and cross-sectional interviews with national established surgical leaders will be conducted.

### Study setting

The study will be conducted across six NHS hospitals that routinely deliver elective orthopaedic services in the NHS, specifically joint replacement surgery of the hip and knee. We aim to purposefully sample the six NHS hospitals according to their performance of these two surgeries, e.g., high versus low performers. In the NHS, patients who undergo joint replacement operations complete patient-reported outcome measure (PROM) questionnaires such as the generic EQ-5D [45]. PROMs are useful in determining the average difference in health status before and after operations and can act as a proxy for hospital performance. In a recent report, Appleby (2020) demonstrated that hospitals could be ranked according to their reported health outcomes and found that 26 hospitals in the UK (12%) reported health outcomes that were significantly better (*n* = 13) or worse (*n* = 13) than the national average [46].

It is important to note that average figures for health gain are adjusted for case mix, which tends to conceal variation amongst hospitals and patients. In this study, we set out to intentionally investigate this variation across hospitals, as some providers appear able to produce better health outcomes for patients than others, even after adjusting for differences in patients and their conditions [46]. We seek to investigate differences to test our theoretical assumptions around DL, and the relationships between implementation leadership and service improvement. To pursue variation in our sample, we will use PROM summary data (2019–2020) for hip and knee replacement to purposely sample three groups of hospital providers [45]; those that are statistically better (group 1, *n* = 2) and statistically worse (group 2, *n* = 2) than the national average (group 3, *n* = 2).

This sampling approach aims to generate a range of NHS service providers in which to explore leadership and the implementation of evidence-based interventions. The first three hospitals we sample (from groups 1, 2 and 3) will form our in-depth exploratory sites. The remaining three with act as validation sites, where we progressively focus on the most important issues of theoretical interest which emerge from our initial analysis [47]. This approach will allow us to ask more focused interview questions as we progress through the later years of data collection and interview the same participants over time [48].

### Component I: embedded social network analysis

We will conduct a social network analysis (SNA) to explore and map how networks of surgeons and staff working in each of the six hospitals are connected and how these networks are associated to the leadership configurations and approaches to implementation [49]. This analysis will enable us to systematically map and analyse the relationships and flows of information between the participants, groups and hospitals systematically and to generate a graphical analysis of their relationships [50]. SNA is based on the idea that the structure of relationships among actors are associated with several outcomes such as learning, creativity, innovation, performance and practice changes [51, 52].

In each of the six hospitals sampled, we will map the social networks in orthopaedic surgery to explore the density and connectivity which is present within and beyond the department. Networks have distinctive topological structures that are associated with different opportunities and constraints to acquire, exchange and apply knowledge [53]. The overarching network structure (e.g., dispersed, centrally concentrated) will reveal the links between surgeons in each hospital and represent how information is exchanged among individuals, for example who and where early career surgeons look to for information [54]. Social networks have important consequences for leadership, knowledge sharing and performance in healthcare settings [55]. In conducting the SNA, we will seek to determine how the groups of surgeons, i.e., the networks, are working and leading as a collective or not, and whether and how these networks are associated to the leadership configurations and approaches to implementation.

SNA has previously been used to identify existing and prospective patterns of collaboration, which can be used to improve knowledge sharing initiatives [56]. Therefore, we will use SNA to identify and compare networks of leadership in each of the six hospitals to inform our understanding of the leadership configurations which are present in hospitals. We will identify the unique characteristics of each of the six hospitals and determine if and how the distinct leadership configurations influence implementation practices. We will seek out interesting points of similarity and difference which may help promote collaboration between surgeons and their professional networks, and the sharing of information and its association with implementation.

### Data collection and processing

Each of the interview participants across the six hospitals will be asked to complete a social network survey during their first (longitudinal participants) or only (cross-sectional participants) interview. Data will be collected at the same time the interview is performed using Qualtrics survey software, version XM (Qualtrics, Provo, UT 2005) [57] available on a tablet, a paper version will be available if necessary. The survey will ask each participant to list the name and role of up to five of the key colleagues in their network. We will explore the participants’ prior relationships with each colleague and how they work together. We will examine how the participants communicate within and across their professional groups and organisation and the extent to which their colleagues provide them with information and advice regarding leadership and implementation. We will ask questions to understand whether the participants believed this colleague was influential in their training and development and whether they trust and talk openly to this person about work. Finally, we will ask whether they believed colleagues hold a shared understanding of the service improvement goals of their department and organisation and whether this relates to the implementation of evidence-based recommendations.

### Data analysis

The data analysis for the SNA will focus primarily on the presence or absence of a relationship between participants working in each hospital. We will examine the relationship or ‘tie’ between two participants, rather than the individual attributes, for example their job role. The presence of a tie specifies the nature of existing relationships, whereas the absence of a tie indicates the potential for relationships to be formed between actors and groups [56]. The networks size will be measured by the number of nodes (participants) and number of ties (relationships) among those nodes. In our study, each node will represent a participant from each of the six hospitals and the colleagues they nominated as collaborators in their working group/professional network. Each tie signifies the presence of a connection between the nodes. Our analysis will enable us to conceptualise and visualise the surgical networks in each hospital and demonstrate how networks of leadership form in each organisation. We will examine the whole network structure in each of the hospitals, for example the overall configuration of leadership in the organisation, in addition to each individual participant networks. The findings from the SNA will illustrate the size, structure and features of leadership networks to identify distinct differences and similarities across hospitals. This will inform and supplement the qualitative data analysis to help illuminate and integrate the narratives of the participants. We anticipate that the connectedness of the networks will provide insight into the leadership configurations in each hospital, i.e., what does leadership look like for each organisation, resulting in an overarching representation of implementation leadership in each of the six hospitals.

### Component II: qualitative interviews

#### Longitudinal interviews

Semi-structured interviews will be conducted with orthopaedic surgeons and their professional network to explore how surgeons learn about best practice and leadership configurations. Questions will explore perspectives of participants regarding medical education, continuing professional development and the influence of leadership and mechanisms of DL on evidence-based practice. The longitudinal interviews will elicit the values and views participants’ hold regarding evidence-based practice, how clinical leadership (both their own and others) influences practice and importantly how their views change and develop over time. The longitudinal design will provide a unique representation, as it aims to tracks changes or lack of changes in participants’ narratives as they undergo professional training and are exposed to different leaders and different approaches to leadership. Longitudinal topic guides will draw on the extant DL literature and an ongoing realist review of leadership configurations in surgery which in being conducted by the research team. Two surgeon members of the study advisory group will pilot interview topic guides to check questions are clear and meaningful.

#### Characteristic of participants and schedule

Interview participants will be purposively sampled from each of the six hospitals identified via the surgery performance ranking. We aim to sample a range of staff as identified in the network and a range of orthopaedic surgeons in each hospital according to career stage and where possible, gender and ethnicity. Here, we define early career stage as orthopaedic specialty trainees and substantive post consultants (between 0 and 15 years since starting orthopaedic training). We will seek to recruit the key participants in each network to form the longitudinal study sample (approximately 4–6 participants per site). However, attrition over time will dictate final numbers of participants. In order to generate longitudinal data, we will conduct an initial interview with these participants and then follow-up interviews every nine months for a 36-month period, thereby interviewing each participant four times. Each participant will provide fully informed consent to take part in the four interviews. Interviews will be digitally audio-recorded and transcribed by a professional transcription company. We understand that maintaining flexibility and perseverance in data collection processes will be paramount in enabling the collection of longitudinal data.

#### Cross-sectional interviews

To understand leadership from the perspective of current surgical leaders, we will conduct semi-structured interviews with members of the surgical leadership team in each of the six hospitals. In our previous exploratory study, we found that leadership in surgical communities was a vehicle which enhanced the mobilisation of evidence-based knowledge in orthopaedics [23, 39]. Therefore, it warrants further investigation to determine the mechanisms at play and the impact on service improvement. Adopting this cross-sectional interview approach will enable us to identify surgical leadership as specified by other members of the surgical team. We will target those actors whom the earlier career stage surgeons and staff believe represent leadership in their organisation, particularly leadership for improving evidence-based practice and innovation. The aim of the cross-sectional interviews is to identify similarities, differences, models of leadership and mapping areas of good practice within and across each of the six hospitals.

To provide a national perspective of surgical leadership and the implementation of evidence-based interventions, we will conduct further cross-sectional interviews with senior members of the orthopaedic community. We will explore the relationship between performance, clinical leadership, scientific evidence and further education in surgery from the leaders’ perspectives. Across the range of cross-sectional interviews, we will gather professional narratives about the influence of leadership processes in post initial qualification training, and how it is configured and whether leadership can act as a mechanism to enact change across organisations, teams and individuals. To explore the potential gender imbalance lack of diversity in surgical leadership, we will purposively sample, where possible, according to gender to ensure senior representation across gender identities ethnic groups. Cross-sectional topic guides will draw on the preliminary findings of longitudinal interviews in the three exploratory sites and progressively focus the data collection as we build narratives around DL, and how and if leadership configurations can change. Consenting, recording and transcription processes will align to those used in the longitudinal interviews.

#### Characteristic of participants and schedule

Cross-sectional interviews at hospital level: Over 12 months, we will interview approximately 18–20 local leaders identified by the surgeons and staff and purposively sampled from the six study hospital sites. We anticipate that these participants are likely to be later stage clinical consultants with more than 15 years’ experience in the field. However, it is possible that it could be any member of the surgical team or social network.

#### Cross-sectional interviews at national level

To obtain a nationwide perspective, we will interview national established surgical leaders, for example the Chair of professional surgical societies (*n* = 10). These participants will be opportunity snowball sampled from the larger network of the research team and during national meetings, professional conferences and surgical leadership training programmes attended by the research team. The study team and advisory group will facilitate additional recruitment of national orthopaedic leaders from organisations such as the Royal College of Surgeons and the British Orthopaedic Association.

#### Data processing

Pseudonymised demographic data will be collected from all participants to provide a descriptive and contextual picture of the participants working in each of the six hospitals, for example demographic details such a surgeon years in post and additional qualifications will be sought. Personally identifiable data used in the data pseudonymisation process will be collated in a password protected database which will be stored to enable member checking later in the course of analysis. In total, we anticipate conducting approximately 120–140 interviews over the course of the 5-year study. During the longitudinal data collection, we will seek to obtain a full dataset from each individual participant, hospital and year. During the cross-sectional national surgeon leadership interviews, it is possible that data saturation will be reached before the maximum number of planned interviews are conducted (*n* = 10). All interviews will be audio-recorded, professionally transcribed and processed in NVivo v12 software (QSR International 1999) [58].

#### Qualitative data analysis

To extend our previous work and determine how leadership can enhance the use of evidence in surgical practice [23, 39], we will perform an overarching analysis of the data adopting a constructivist grounded theory analytical approach [59]. Constructivism takes context, beliefs and actions into account and assumes the existence of multiple experiences, both between the researcher and the multiple participants [60]. Charmaz (2000, 2001) [61, 62] emphasises the need to maintain participants’ words and language in the analytical process; thus, it is necessary to preserve the participants presence throughout data analysis and reporting [63]. The constructivist grounded theory approach fits well with the study aim, as it enables the co-construction of meaning between all study actors, whilst simultaneous taking account of the reflexivity of the research team [64].

**Fig. 1:**
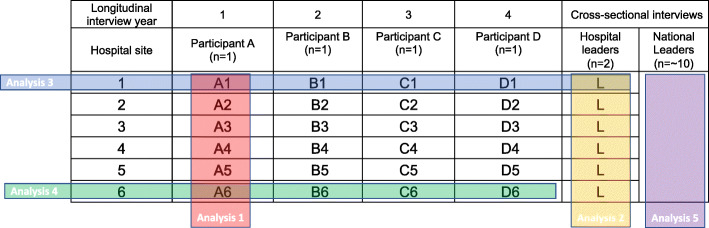
Data analysis matrix

Constructivist grounded theory entails an ‘imaginative engagement with data’ and a focus on flexibility as analysis progresses [65]. The fluid framework proposed by Charmaz contains at least two stages to coding of the raw data (initial or open coding, then refocused coding [identifying the codes that are recurring or particularly significant in illuminating]), followed by construction of theoretical categories through to a grounded theory, using techniques such as memo writing, constant comparisons and theoretical sampling [65]. During data collection, we will maintain a reflexive account of both the data collection and analysis processes to ensure consistency. Consistency is imperative during long periods of longitudinal data collection. To maximise the explanatory power of our findings, we plan to segment our analysis across different levels of data analysis, examples of these analysis are depicted in (Fig. 1)

To assess variation in the approaches to leadership across the hospitals, we will analyse data within each study year by hospital location (sites 1–6). To explore temporal factors, we will analyse data across data collection years 1–4 (analysis 3 network participants plus surgeon leaders, analysis 4 network participants only). To provide a conceptually and contextually nuanced framing of surgical leadership, we will analyse the individual hospital leader data by site (*n* = 6) (analysis 2) and the national surgical leader data (analysis 5) independently and in comparison to each other to extend the depth and breadth of our study findings. Therefore, the novelty of our study not only lies in the longitudinal qualitative design, but also in the explanatory power generated through the complexity and extensiveness of the planned analysis.

## Discussion

The implementation and use of evidence-based interventions in orthopaedic surgery is an important part of healthcare practice to help drive service improvement [2]. However, across surgery, we lack empirically grounded implementation strategies and interventions which can be targeted at those individuals, professional groups and organisations seeking to make improvements. In this paper, we have outlined our research question, study aim, theoretical and methodological approach. The findings of our research will have duel benefit for practitioners and researchers concerned with evidence-based implementation in surgery. First, the detailed methods planned and described will enable us to unpick the features and ingredients of successful leadership for evidence-based implementation, service improvement and innovation. This will generate the empirical and theoretical data needed to develop leadership interventions that are grounded in the study findings and aimed at generating service improvements.

In healthcare, it can be difficult to untangle the concepts of leadership, performance and investment; therefore, we set out to examine these in parallel by collecting data from a range of hospitals to intentionally investigate variation in performance, using published outcomes for elective hip and knee surgery as a proxy. We recognise that there is often a lag between leadership and its effect on performance and that an effect may be due to factors other than leadership (more resources for example). Therefore, where possible, during the interviews, we will explore whether our outcome measures are linked to an intervention that was implemented and/or whether clinical practice has fidelity to the evidence base.

Second, we will extend the literature on leadership in implementation research by revealing the configurations of leadership which appear to be effective in getting evidence into practice. Throughout our study, we position leadership as the contextually situated process of a collective group, not the actions of the person who attends leadership development programmes. The leadership networks mapped in this study will be illuminated and enriched through the narratives of participants to understand the leadership configurations that exist in practice and those which appear more effective for improving implementation of evidence-based practice. Thereby answering the questions, how do you get leadership for improvement? Which actors are best placed to perform leadership to improve implementation? What do these actors need? And what are the organisational conditions that allow it to happen?

We have positioned our research to critique existing notions of healthcare leadership which have focused on individualistic heroic leaders, who lead independent of context. This is a prominent issue within the surgical professions, where there is evidence of, and support for, individual hierarchical leadership approaches. Professional surgical societies promote generic aspects of individual leadership, through training in subjects such as situational awareness, communication and teamwork [20, 66, 67]. However, existing surgical leadership courses make little explicit reference to scientific evidence, or its implementation in training and clinical practice. Leadership and management training is now required as part of surgical training; however, there is little standardisation or requirement for content and therefore, a lot of variation in delivery [68]. The lack of national policies and training investment means that leadership practice remains uneven and unfocused on implementation of evidence-based interventions [69]. We also cannot guarantee that healthcare organisations and systems are set up to support the output of leadership development programmes. Consequently, ‘leaders’ may not always have the power and disposition to enact leadership at scale or to achieve sustainability, particularly when it has implications for other disciplines working in healthcare.

### Strengths and limitations

The key strength of our research is the use of multiple sources of data (longitudinal and cross-section interviews and SNA) to study the same leadership phenomenon. The combination of these multi-component, multi-site, multi-agent methods will enable us to overcome the weakness that extend from using a single approach [70]. If the data from different methods conflict, or appear as negative cases or illuminating insights, this will provide us with an interesting opportunity to investigate the meaning behind the differences. For example, we may identify unexpected nodes in the SNA, who represent individuals or groups who are central and influential to leadership processes in the hospital. The comparative process of data collection and analysis both within and across hospitals will lead to a more sophisticated understanding of our data [71, 72]. Integrating the quantitative and qualitative, so that both the processes and impacts of leadership may be investigated, is necessary to address the study aim and enhance the credibility of our findings and contribute to the explanatory power of our work.

Our planned study has several limitations which reflect the practical and operational issues we may face during the data collection. These mainly concern the initial recruitment of hospitals and interview participants, but then the follow-up and attrition of study participants in a longitudinal qualitative study (i.e., over 4-year timepoints). We aim to sample and recruit six hospitals from a population of 204 in England (3 exploratory and 3 validatory). Therefore, if we fail to recruit our targeted hospitals, we will select the next hospital listed in each PROMs performance category. If we fail to recruit a range of professionals/professional grades/surgical leaders in a hospital, we will re-evaluate the sample to achieve a balanced mix of participants.

We anticipate further recruitment challenges in achieving our aim to ensure representation across gender identities and ethinic groups. The UK has a longstanding imbalance in the make-up of the surgical consultant workforce, with females comprising only 7% [73]. Access to and recruitment of a range of participants will need constant consideration and monitoring during the recruitment phases of the study. We aim to maximise our sample and minimise participant attrition by establishing clear communication channels and offering flexibility to the participants during data collection where possible.

A final limitation is the self-report nature of the SNA survey which may lead to biased responses. Completion of the survey will also be done in the presence of the research team which may drive idealised responses regarding who participants suggest represent leadership in their organisaitons [74]. To ensure the quality and rigour of our data, we will explore and validate the results of the SNA during the interviews and conduct crosschecks across participants working in the same organisations. During the network analysis, we will make the assumption that the networks produced for each hospital are standardised across time and will remain static for the period of longitudinal data collection. However, it is possible that individuals in the network leave the organisation or change roles. Where possible, we will seek to uncover details regarding any changes and the impact they had during the interviews. The limitations of the methods used will be taken into account in the interpretation of the study findings.

## Conclusion

The aim of our research is to understand and explain how differing configurations of clinical leadership are enacted and how they influence the implementation of evidence-based practice. We will seek to understand how approaches to leadership (i.e., distributed, individualistic) can be improved to enhance the use of evidence in elective orthopaedic surgery in the NHS. The longitudinal and cross-sectional interview data, in combination with the output of the SNA conducted in each hospital, will allow us to understand what leadership configurations are present across a range of organisational healthcare contexts. The results of our study seek to establish the connexions between contextual DL and its links to evidence-based outcomes in surgery, which can be achieved through the cumulative interactions of multiple actors across the organisation.

Our analysis will enable us to understand how and why different configurations of leadership develop and under what organisational conditions they are able to flourish. In doing so, we critique existing notions of implementation leadership, which portray leadership through the behaviours and attributes of individual champions working within distinct clinical communities. We adopt a DL approach as the conceptual and theoretical framework guiding our study, to explain leadership as a collective endeavour which cannot be separated from context. The study findings will, we hope, guide the development of interventions that are grounded in the data and aimed at advancing leadership for service improvement and innovation which we will implement and test in future research.

## Data Availability

Data sharing is not applicable to this article as no datasets were generated or analysed during the current study protocol.

## Abbreviations

DL: Distributed leadership
EBP: Evidence-based practice
PROM: Patient-reported outcome measure
SNA: Social Network Analysis
